# Quantifying empirical support for theories of consciousness: a tentative methodological framework

**DOI:** 10.3389/fpsyg.2024.1341430

**Published:** 2024-03-14

**Authors:** Asger Kirkeby-Hinrup

**Affiliations:** Department of Philosophy, Lund University, Lund, Sweden

**Keywords:** consciouness, inference to the best explanation, Bayesian updating, empirical evidence, theories of consciousness, assessment, comparison

## Abstract

Understanding consciousness is central to understanding human nature. We have competing theories of consciousness. In interdisciplinary consciousness studies most believe that consciousness can be naturalized (i.e., consciousness depends in some substantial way on processes in — or states of — the brain). For roughly two decades, proponents of almost every theory have focused on collecting empirical support for their preferred theory, on the tacit assumption that empirical evidence will resolve the debates. Yet, it remains unclear *how* empirical evidence can do this *in practice*. Here I address this issue by offering (a sketch of) a methodology to quantify the divergent sets of empirical support proposed in favor of extant theories of consciousness. This in turn forms the foundation for a process of inference to the best explanation inspired by Bayesian confirmation theory. In interdisciplinary consciousness studies we are blessed with an abundance of theories, but we have reached a point where, going forward, it would be beneficial to focus on the most promising ones. Methods for assessment and comparison are necessary to identify which those are. While future refinement is likely, the methodology for assessment and comparison proposed here is a first step toward a novel way of approaching this through a quantification of empirical support for theories of consciousness.

## 1 Introduction

The field of interdisciplinary consciousness studies (ICS) — i.e., work at the intersection between philosophy of mind, psychology, cognitive science, and neuroscience — has been blossoming over the last decades. Yet, the current state of the field of ICS is precarious, and further development is necessary. In other words, we do not want to remain forever in the current stage of our field, in which we have dozens of theories and no noncontentious way of deciding between them. A positive upshot of this issue has been several proposals of how to assess and compare theories. The (sketch of a) methodology I offer in this paper is a novel proposal for this.

ICS converges (approximately) on the belief that understanding the brain’s role in relation to consciousness is central to understanding consciousness *per se,* as well as its associated concepts (e.g., experience, cognition, meta-cognition, emotion, action, and perception). As Weisberg (2014, p. 433) writes: “[…] rooted in empirical data. This is the proper way to approach consciousness.” Weisberg is not alone in this sentiment. In ICS the shared assumption is that empirical data carries evidential weight in determining the plausibility of a theory of consciousness. But how do we compare the evidential weights of the competing sets of empirical evidence proposed in favor of extant theories of consciousness? Most theories of consciousness on the market are internally consistent conceptual frameworks that propose mechanism (s) underpinning phenomenal consciousness (Doerig et al., 2020 for a useful classification of the different kinds of proposed mechanism; see also Sattin et al., 2021; Signorelli et al., 2021; Schurger and Graziano, 2022). Presently, the field of consciousness studies offers a wide variety of theories [e.g., the Global Workspace Theory of Baars (1996); the first-order theory of Block (1995); the Dispositional Higher-order theory of Carruthers (1998); the Same-Order Metarepresentational Account of Cleeremans et al. (2020); the Global Neuronal Workspace Theory of Dehaene and Naccache (2001); the Predictive Processing Theory of Friston (2013); the Wide Instrinsicality View of Gennaro (1996); the Same-Order Monitoring theory of Kriegel (2007); the Recurrent Processing theory of Lamme (2004); the Attention to Intermediate Representation theory of Prinz (2005); the Higher-Order Thought theory of Rosenthal (1997); the Integrated Information Theory of Tononi et al. (2016); the Higher-Order Global state theory of Van Gulick (2004), to name a few]. While they can be grouped into different ‘families’, they mostly offer mutually exclusive explanations of the structure and function (s) of consciousness (at least they supposedly do. For further discussion see Kirkeby-Hinrup et al., 2023).

Broadly speaking, the questions related to consciousness fall into two distinct domains: the first concerns information processing and behavior (cognitive domain); the second concerns the experience of being — or *what it is like to be* (Nagel, 1974) *—* conscious (phenomenal domain). Current theories largely agree about the cognitive domain, at least with respect to functional characteristics and behavioral predictions, but they differ with respect to the phenomenal domain. In fact, a major fault line in the debates between theories of consciousness concerns the nature and importance of phenomenality (i.e., what-it-is-like to be conscious). This question roughly divides the field into two camps: proponents of *deflationary* accounts (Rosenthal, 2008, 2012) and those who advance *inflationary* accounts (Block, 2011b). The latter sees phenomenality as widespread in — and central to — consciousness, whereas the former denies this. Yet, both deflationary and inflationary accounts tend to use the same vocabulary, a problem noted by Rosenthal who says: “The phrase ‘what it’s like’ is not reliable common currency” (Rosenthal, 2011, p. 434). When competing theories each are internally consistent, describe the target phenomenon using many of the same concepts — yet disagree about what those concepts actually mean — there is little avenue on conceptual grounds to determine which theory is correct, or even preferable. This has left the conceptual debate largely gridlocked because it is difficult to criticize a theory without begging the question against its underlying conceptual framework. Thus, it is unclear at best if there is an avenue forward in arguing about consciousness solely on conceptual grounds.

However, because most people involved in these debates share the assumption that consciousness can be naturalized (i.e., consciousness depends on physical processes, assumed to occur primarily in the brain), the hope is that empirical evidence may resolve these disagreements by determining which theory is more empirically plausible. Consequently, in recent decades there has been a radical increase in the application of empirical evidence in support of — or to argue against — theories of consciousness (c.f. Yaron et al., 2022, p. Figure 2b). Proponents of most theories have advanced empirical evidence to illustrate its explanatory power, and/or scaffold its claim to plausibility on a general level. This is reasonable standard scientific practice, and overall a good approach. However, in the last couple of years, attention has turned to how — or whether — empirical evidence actually may do the work for us we hoped it would (determining which theory is most plausible/preferable). This attention has illuminated many issues with respect to how we collect, deploy, assess, and compare empirical evidence in ICS, as often cast in light of well-known considerations from the philosophy of science (Seth, 2009; Del Pin et al., 2021; Kirkeby-Hinrup and Fazekas, 2021; Overgaard and Kirkeby-Hinrup, 2021; Schurger and Graziano, 2022; Kirkeby-Hinrup, 2024). These issues pertain to whether — or how — empirical evidence can help us decide which theory is ultimately most plausible/preferable on a long-term perspective (i.e., which theory is closest to truth(s) about the world with respect to propositions about the phenomenon which we call “consciousness”). Furthermore, even on a short-term perspective do questions about the work empirical evidence can do for us appear. The current abundance of competing theories in ICS can only be a positive thing if there is a way to eliminate theories as part of our scientific process of approximating the truth.

I will, in the next section, examine two existing proposals to gauge the state of the field and set the appropriate context. One — based on *criteria* — proposed by Doerig et al. (2020)consists in assessing and comparing theories according to their explanatory scope and ability to handle principled problems. The other endeavor is of strictly empirical nature and turns on the notion of *adversarial collaboration,* i.e., getting proponents of competing theories to agree on an empirical paradigm on which their theories have differing predictions, and then performing the experiment.^1^ In section three, I introduce the general context for my proposal, before presenting the details in section four. Finally, in the fifth section, I offer some concluding remarks.

## 2 Comparing theories of consciousness

How do we — based on empirical evidence — determine which theory of consciousness is preferable? Currently, there are two prominent approaches to this question (this paper proposes a third). The first approach operates on a principle similar to falsification. The second approach deploys a set of criteria to assess and compare theories of consciousness. Briefly considering each of these is appropriate here because understanding the strengths and/or shortcomings of existing approaches provides anchors for evaluation of the third approach I will present in sections three and four. Consequently, let us consider these in turn.

The first approach, operating on the principle of falsification, consists of a range of separate projects, and is called “Accelerating Research on Consciousness” (ARC). This enormous and ambitious project rightfully has drawn significant attention and praise in ICS. The methodological approach in ARC is the principle of adversarial collaboration, i.e., testing specific paradigms (agreed upon in advance by proponents of each theory) where competing theories predict different (supposedly concrete and mutually exclusive) empirical measurements. The results of each project are then taken to strengthen the theory whose prediction is confirmed, and (partly) falsify the other(s).

Recently, (Ferrante et al., 2023; Melloni et al., 2023) the results of the first project in ARC have been made public. In this project, predictions of Integrated information theory (IIT) (Tononi et al., 2016; Albantakis, 2020) was compared to those of the Global Neuronal Workspace theory (GNWT) (Mashour et al., 2020). The results were unclear, neither fully supporting either theory, nor fully falsifying either theory. Consequently, in terms of eliminating theories, or assessing which is preferable to the other, the first ARC project did little to move the needle between IIT and GNWT. In subsequent debate, proponents of both theories point to limitations in the data and reach opposing conclusions regarding the involvement of the prefrontal cortex (Ferrante et al., 2023).

However, even if this ARC project had provided — or if the next projects in ARC provide — more conclusive data, another problem remains. The problem is that it is standard scientific practice to revise theories in light of new evidence. So, failing to have your predictions confirmed is likely to be taken as an incentive to further develop a theory, rather than abandon it. That is; proponents of a theory are not immediately inclined to completely abandon a theory if it comes out unsuccessful in an ARC project. To boot, we do not know what the ‘threshold’ for amount — or quality — of evidence is for a theory to be abandoned. Put differently, it is unclear how many — or which kind of^2^ — ‘losses’ on ARC projects are sufficient for a theory to be abandoned by its proponents. Problematically however, there is a real risk that this may arbitrarily depend on the individual proponents of a theory. This may raise worries about whether ARC ultimately will be able to deliver results with the requisite ubiquity to falsify a theory to the extent that it is eliminated from further consideration by the field (this worry was echoed by Lucia Melloni when presenting the aforementioned first results of the ARC project at the 2023 ASSC conference in New York with the words: “No one changes their mind” with reference to the Daniel Kahneman, the originator of the adversarial collaboration idea, who had declined to participate in the presentation for that reason). In the long term, whether ARC will be able to change minds remains to be seen, but (assuming an interest in consciousness) it would certainly be in everyone’s interest if it can. Now, in addition to these overall worries (that apply to any way of assessing and comparing theories, including the one proposed below), there is a range of more concrete issues — of either a methodological or practical nature — with ARC. Call the first of these: *targeted theories*. ARC projects inherently treat only a subset of the theories (between two and four theories per project currently).^3^ This means ARC can never say something about the field as a whole, but only about some specific relation between a few theories and some specific data. The second issue is that ARC has a *narrow scope*, in the sense that each comparison is based on one or a few paradigms.^4^ Barring some auxiliary framework, this restricts conclusions to the results of the few paradigms, precluding conclusions about overall plausibility.^5^ A third issue is methodological *generalizability*. There are two sides to this issue. The first side is practical, and derives from the fact that, in ARC, paradigms and pipelines deployed to test theory A and B, cannot be applied to test theory C and D. This makes ARC very (time, expertise, money) cost intensive. The second side is methodological; because we are not in a situation where one paradigm ‘fits all’, it is unclear how to compare results from different ARC projects. For instance, if project 1 confirms theory A over theory B, and project 2 confirms theory B over A, which should we prefer?^6^ The fourth issue concerns the *robustness* of ARC results. Because of their specificity, the results from ARC are very sensitive to changes in theories. Therefore, if (aspects of) theory A is revised to account for a failed prediction in an ARC experiment, this will require a whole new ARC project to assess the revised version of the theory. Since revising theories in light of new evidence is standard scientific practice, one would expect such revisions to happen. Finally, the fifth issue is the *cost* of ARC. In line with its ambitious and comprehensive approach (Ferrante et al., 2023; Melloni et al., 2023) the current ARC projects require significant human, financial and institutional resources. On the one hand, this speaks to the scientific rigor, ambitiousness, and effort of ARC. On the other hand, the cost of ARC is prohibitive to the vast majority of researchers in the field, which means it is unlikely to be broadly adopted. The previously discussed issues of *generalizability* and *robustness* further compounds the *cost* issue, since every time a theory is revised (*robustness,* for instance due to results from an ARC project) we need a new tailor made (due to *generalizability*) multi-year multimillion dollar project to assess the new version. This is a steep cost and should raise worries about the long-term feasibility of the ARC approach (especially, if we do not even know what it would take for someone to change their mind).

The second major approach consists in developing and deploying a set of criteria to evaluate and compare theories. The criteria based approach (CRIT) has been advanced by Doerig et al. (2020). They propose two categories of criteria for assessment (e.g., table in Doerig et al., 2020, p. 48). The first category, they dub *criteria*. This category consists of four challenges a theory of consciousness may face depending on the hypothesized mechanisms underpinning consciousness. The second category Doerig et al. call *scope*. Here, they propose to deploy five classical distinctions about consciousness to assess which aspects of the phenomenon are covered by a given theory. CRIT has already been the subject of much debate (Doerig et al., 2021). Here, I highlight four issues that are of particular relevance in the present context. The first of these issues concerns CRIT’s *sensitivity* to empirical evidence. The issue is that CRIT ignores the amount of empirical support of theories outside of satisfying criteria, or the amount of empirical evidence a theory’s meeting of a criterion relies on. While many of the proposed criteria are framed against an empirical background, CRIT only superficially takes into account actual empirical evidence proposed in favor of theories. This means a theory with a lot of empirical support will be scored as equal to a theory with almost no empirical support as long as they satisfy the same criteria. Similarly, CRIT does not take into account the amount of empirical evidence a theory’s meeting of a criterion relies on. Theory A is scored as equal to theory B as long as they satisfy the same number of criteria, regardless of their respective sets of evidence. The second issue concerns *arbitration* between theories. Suppose two or more theories satisfy the same number of criteria, how do we decide between them? Given the limited number of criteria and the limited grading system on each criterion (e.g., table in Doerig et al., 2020, p. 48), the possibility of ties is high. *Arbitration* concerns not only how to decide between two or more theories that satisfy identical sets of criteria, but also how we should decide between two or more theories that satisfy the same number of criteria without their sets being identical. In other words, we need to know how to weigh satisfying criterion A against satisfying criterion B. CRIT is certainly useful for an overall classification of theories, but because it is not sensitive to divergent amounts of support, it is insufficient for any fine-grained comparison of theories. The third issue concerns the *flexibility* of the criteria. Now, Doerig and colleagues are explicit that the current set of criteria is not intended to be exhaustive (Doerig et al., 2020, p. 42) and will likely need expansion.^7^ But how many — and which — criteria can we add? One might hope that the answer to this question is that any further criteria will be obvious, and we will come upon all — or most of — these over time (which in turn limits the maximum possible number of criteria as well). Observe, this answer may lead to a debate about what “obvious” entails, to whom it will be obvious, and who gets to decide these questions. This is the fourth issue: *arbitrariness*. For now, I will leave *arbitrariness* to the side since this issue will loom large throughout the text, and instead focus briefly on another upshot of *flexibility*; namely the question of how many criteria we will need to distinguish convincingly between theories (assuming we even can do this in a non-arbitrary way). Presently, any speculation on an exact number of further criteria would be premature. But given that the present set of criteria makes ties likely, it is likely to need expansion in the future. The next thing to note is that the set of criteria that are theory-neutral, obvious, overarching, and important is likely limited (however see, Rosenthal, 2021). This limitation would make any further criteria less central than the nine currently proposed. One reason for thinking this is that, if there were indeed further obvious and important criteria, Doerig and colleagues would have included them in their paper.^8^ Be that as it may, it nevertheless is likely that a future expansion of CRIT will result in increasingly detailed criteria of less and less importance. One positive upshot of adding more criteria is that it seems CRIT may be able to deal with *arbitration* since ties will be less likely^9^ as the number of criteria increases. However, this at the same time would undermine the main appeal of CRIT, i.e., identifying the *overarching principled* criteria a theory of consciousness should satisfy.

In the rest of this paper, I will present a third approach to assessing and comparing theories based on the notion of inference to the best explanation (IBE). Importantly, while I have identified shortcomings of both ARC and CRIT (*targeted theories, generalizability, robustness, cost* and *sensitivity, arbitration, flexibility, arbitrariness*, respectively), and will show that the approach proposed here does not have these shortcomings, I am not advocating that ARC and CRIT have no value, let alone should be given up. The approach here is intended to complement, rather than supplant, ARC and CRIT. There is room for these three approaches, not only to coexist, but to develop also a positive synergy. I will return to this in the concluding remarks.

## 3 Inference to the best explanation

In the previous section, I discussed two contemporary approaches to assessing and comparing theories. Previously, together with Peter Fazekas (Kirkeby-Hinrup and Fazekas, 2021), I have advocated a third approach based on the notion of inference to the best explanation. Looking at the publications over the last couple of decades, an IBE process seems tacit in much of the work concerned with the relation between empirical evidence and theories of consciousness. One of the most explicit invocations of IBE can be found in the work of Ned Block (2007, p. 486) when he says: “I have in mind […] the familiar default ‘method’ of inference to the best explanation, that is, the approach of looking for the framework that makes the most sense of all the data […].” Yet, to my knowledge, outside of my proposal with Fazekas, no one has endeavored to attempt inference to the best explanation in practice in ICS. One reason may be that classical versions of IBE are ill-suited for straightforward application in our situation.^10^ This is because we cannot compare theories on their explanatory powers, because there is no consensus on a common explanandum. To elaborate, competing theories do not necessarily have identical explanatory targets (Sattin et al., 2021; Signorelli et al., 2021; Yaron et al., 2021), yet are taken to be mutually exclusive for the reason that they all target the same phenomenon (and they share the assumption that there is only one phenomenon). In the vocabulary of Chalmers (2002), theories differ in their ‘intension’ of the explanandum (the meaning of the word ‘consciousness’), but coincide on its ‘extension’ (the thing in the world picked out by the word ‘consciousness’). In a way, when deploying empirical evidence in assessing and comparing theories of consciousness, we are hoping to resolve disagreements on the *intension* through investigations of the *extension.* The upshot is that we cannot adopt explanatory power as our metric for comparison, since explanatory power depends on the ‘intension’ of the explanandum, which means we would be comparing apples and oranges. Therefore, we must perform IBE on the basis of some other metric than explanatory power. One way to approach this is by collating the respective sets of proposed empirical support of the competing theories, to determine if our observations about the *extension* (empirical evidence) conform to a proposed *intension* (a theory), and how well.

The notion of IBE (sometimes understood as co-extensive with the notion of abduction) is a classic topic in the philosophy of science (Burks, 1946; Harman, 1965; Peirce and Hartshorne, 1974; Minnameier, 2004, 2010; Campos, 2011; Douven, 2021). But, for the reason just given, classical notions are not straightforwardly applicable in our case. Therefore, some clarification is necessary with respect to the way IBE is conceived of here. Firstly, the ‘explanations’ we need to infer to are theories of consciousness (what Block called “frameworks” in the quote above. In Chalmers’ vocabulary, the different proposed *intensions* of consciousness). Secondly, the assessment and comparison are not based on *explanatory* considerations, *per se.* The metric for assessment — and what is being compared — is not a theory’s explanatory power in relation to its targeted explanandum (its *intension*). As just noted, comparing explanatory power in relation to *intension* of the explanandum is problematic because there is no agreement on what a good explanation would entail, because there is no agreement on the exact characteristics of the phenomenon (see, e.g., debate in Rosenthal, 2011; Weisberg, 2011; Block, 2011a,c). To avoid this, the IBE approach could consist in assessing and comparing (i.e., inferring on the bases of) the explanatory power of theories in the empirical domain. In other words, the metric of comparison in this proposal is the ability to explain and predict empirical data.

There are many ways to develop an IBE process. Fazekas and I (Kirkeby-Hinrup and Fazekas, 2021) proposed a four-step process relying on the fact that the first step (assimilation, i.e., data collection) was already far along,^11^ argued the importance — and demonstrated the feasibility — of the second and third step: compilation and validation (respectively concerned with compiling the proposed evidence for each theory, and validating claims of empirical support on a case-by-case basis). To elaborate, in addition to demonstrating that the second and third steps are feasible by showing what they look like in practice, the main point of the paper was that if we want to decide between theories on the bases of their respective empirical support, we had better know what their respective empirical support is,^12^ and whether any given piece of empirical support claimed by a theory, in fact supports the theory. The step of the IBE process that is developed below is the one we did not treat in that paper, namely the actual comparison of theories,^13^ the fourth and final step. Since the proposal here depends on quantifying the competing sets of proposed evidence, I will call this approach *Quantification to the Best Explanation* (QBE).

### 3.1 An intuition about weights of evidence

**Figure 1:**
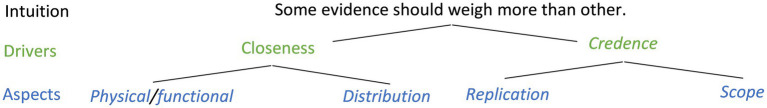
The intuition and two drivers.

An initial desideratum is that QBE should avoid the identified shortcomings of ARC and CRIT (*targeted theories, generalizability, robustness, cost*, *sensitivity, arbitration, flexibility,* and *arbitrariness*) discussed in the previous section. While QBE avoids many of these easily, two warrant consideration here,^14^ namely: *sensitivity* and *arbitration*. These two shortcomings appear to threaten QBE and CRIT equally. To clarify, the sets of empirical evidence proposed for the extant theories of consciousness are *prima facie* incommensurable. One source of the incommensurability is that the sets of empirical support for each of the theories — while in many cases partially overlapping — do not contain exactly the same elements. Thus, the *arbitration* issue reappears on IBE, because we now need a way to weigh the non-overlapping elements against each other. To illustrate, it is unclear whether theory A being supported by the change blindness phenomenon is more “valuable” (as it were) than theory B’s support from the split-brain phenomenon. Yet, many share the intuition that *some* instances of empirical evidence should weigh heavier than others. This raises at least two questions: First: what is the driver of this intuition? And second: which instances? Let us focus here on the first question (the second question will be addressed in subsequent sections). Leaving open whether there are others, here are at least two possible candidate drivers of the intuition that some evidence is more “valuable” (should weigh heavier in IBE) than other (Figure 1).

The first candidate as a driver is that the ‘closer’ (applicable) a piece of evidence (a phenomenon) is to the normal human condition (i.e., consciousness *as such,* or consciousness in neurotypical adults) the higher weight it should be ascribed in a comparison process. Call this the *closeness* driver. Closeness could be understood as *physical/functional* closeness, suggesting that studies with human subjects are more “valuable” than animal studies or computational models. Another example of *physical/functional* closeness could be the intuition that studies on neurotypical brains are preferable to studying very rare cases of brain trauma or cognitive dysfunction. Another way to understand *closeness* could be as *distribution*, which can be subdivided into inter-individually and temporally, where the former tracks the number of individuals to which the phenomenon applies and the latter tracks *how often* an individual or group of individuals instantiate the phenomenon. Accordingly, phenomena with high distribution (inter-individually, temporally, or both) are ‘closer’ to the explanatory target (‘neurotypical adult consciousness’ because *many* experience the phenomenon *often*) and consequently should be given higher weight in the IBE process.

The second candidate driver of the intuition (that some evidence should weigh heavier than other) concerns our *credence* in the evidence in question. Call this the *credence* driver. According to *credence*, the extent of our knowledge of a given phenomenon seems to matter for the weight it should be ascribed in the IBE process. *Credence*, furthermore, can be subdivided at least into *replication* and *scope*. *Replication* concerns the robustness of our ways of knowing about the phenomenon, i.e., the total amount of studies conducted on it, the amount of replication studies, and the existence of well-established paradigms to investigate it. *Scope* concerns the number of angles we (could) have approached the phenomenon from, i.e., the range of empirical techniques (that can be) applied to it. To elaborate; *replication* considers that phenomena, that have been the subject of thousands of studies and on the basic features of which (independent of any specific theory of consciousness) there is a general consensus, are more valuable than phenomena that have only been recorded very few times and the interpretations of which are widely contentious *outside* of consciousness studies. *Scope*, on the other hand, concerns the number of empirical techniques that have been — or could be — used to investigate the phenomenon. According to *scope*, phenomena that have been measured in many ways (e.g., fMRI, EEG, PET, MEG, ECoG, fNIRS, eye blinks, saccades, eye-tracking, D′, Meta D′, reaction time, introspective report, perceptual awareness scale, to name a few) are more valuable than phenomena that only have been — or only can be — measured using a single or few techniques (e.g., phenomena relying solely on introspective report). Thus — overall on *credence* — the intuition would be that phenomena with either high replication, broad scope, or both should be given higher weight in QBE.^15^

## 4 Bayesian inference to the best explanation

If we follow the intuition that some evidence should weigh heavier than other, a second shortcoming of the existing approaches that also is a challenge for QBE is *arbitrariness*. In this context, *arbitrariness* concerns *who* gets to assign the weights to the pieces of empirical evidence, and whether this can be done in a theory-neutral and non-contentious way. The “who” matters because, if the assignment of weights in QBE depends arbitrarily on the person performing the comparison, the objectivity of the process is compromised which, for obvious reasons, would be a bad thing. Consequently, a second desideratum for QBE is that it can deliver an objective way (one that does not depend on arbitrary choices of the person performing the comparison) to ascribe weight to proposed empirical support.

As an starting point, Fazekas and I (Kirkeby-Hinrup and Fazekas, 2021) suggested that— in order to stay neutral between theories when evaluating evidence — it is preferable to evaluate each piece of evidence in light of the conceptual framework of the theory it is applied to. This is because using any other conceptual framework (‘intension’ of consciousness) risks begging the question against the theory, i.e., presuming a viewpoint and thereby giving up on objectivity.

From this starting point, since we are aiming to quantify empirical support, we need a way to get numbers, via the sets of empirical evidence proposed in favor of extant theories. The conversion to numbers is made difficult by the way empirical evidence is proposed in ICS, *viz* what we are trying to quantify is really arguments to the effect that some piece of empirical evidence is predicted by — or can be explained in light of — a given theory. Such arguments, in turn, depend on the conceptual framework of the theory, and the mapping of an interpretation of some empirical data to this framework (see Kirkeby-Hinrup and Fazekas, 2021 for details).^16^ This kind of conceptual work does not allow for straightforward quantification (i.e., conversion into numbers).

**Figure 2:**
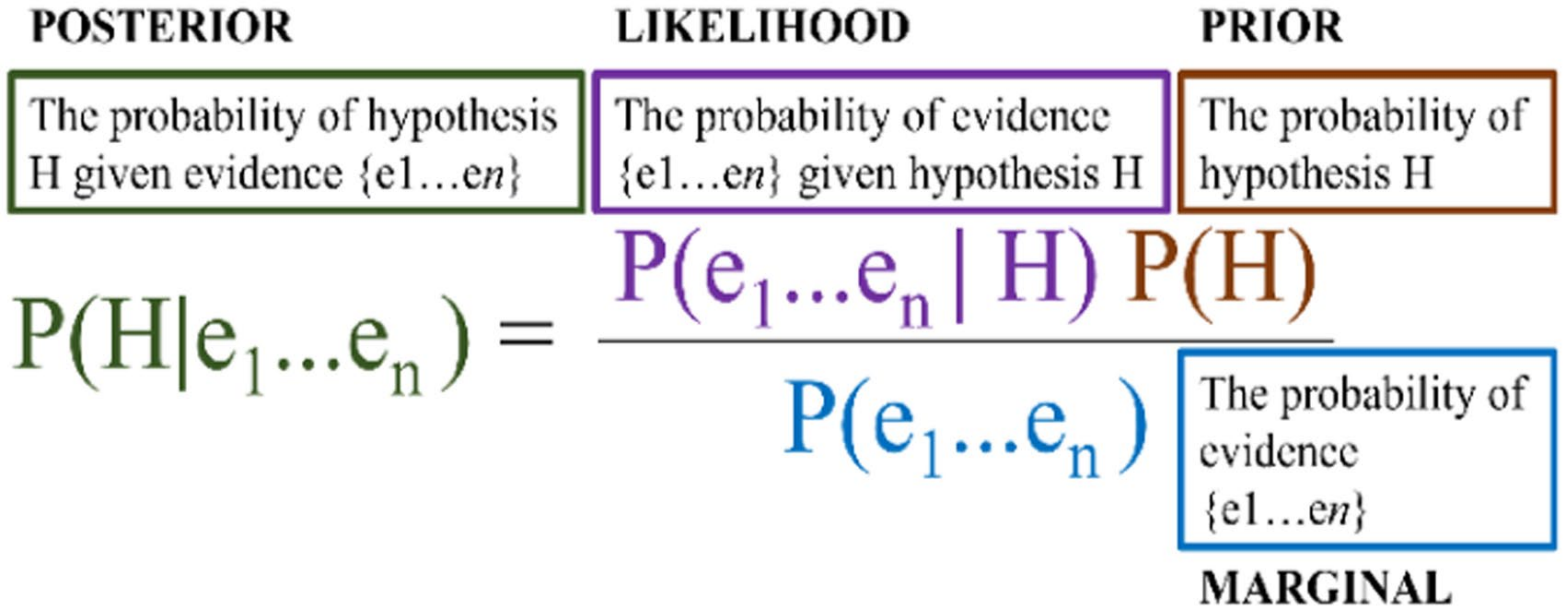
Bayes’ theorem.

Before we turn, in the next section, to solving this challenge, let us briefly consider how the numbers will be used once we have them. The inferential process for comparison that forms the core of QBE takes inspiration from Bayesian Confirmation Theory (BCT) (Gelman and Shalizi, 2013; Crupi, 2021) to estimate the strength of evidence in favor of each theory. A positive feature of BCT is that it delivers a posterior probability for each theory *given* all the evidence proposed in its favor and tells us that we should have more credence in higher posteriors than lower ones. I.e., theories with a high posterior are preferable to theories with a lower one.^17^ The core of BCT is Bayes’ theorem (Figure 2), however, due to the nature of the data QBE quantifies, some accommodation of this is necessary. The rest of this section will be dedicated to clarifying one by one how to reconstruct and understand each of the elements in Bayes’ theorem in the context of QBE.

First and foremost, in order to determine the Likelihood and the Marginal in Bayes’ theorem we need to know what exactly the evidence (e_1_…e_n_) is in the present context. For QBE an “e” is a claim of empirical support. Such claims, in turn, are generally structured as arguments connecting a given empirical phenomenon with a theory of consciousness, aiming to show that — and/or how — the theory can either predict or explain an observation about the phenomenon. In other words, we are dealing with three components: (1) an empirical *phenomenon*, (2) an *observation* about it, and (3) an *argument*.^18^ Let us consider these in turn. *Prima facie*, the class of phenomena invoked by extant theories is very heterogenous allowing many kinds of entries. Examples include pathological conditions such as visual neglect (at varying levels of description, e.g., psychological, behavioral, and physiological), neural processes such as recurrent processes (e.g., as biological, physical, or network-level descriptions), and behavioral phenomena such as visual masking (e.g., as methodology or behavioral descriptions). Consequently, the *phenomenon* concept in IBE must be very inclusive, since limiting the empirical evidence to certain types of phenomena would be arbitrary and risks undesirably biasing QBE against a theory. As a starting point, the *phenomenon* can be conceived of as the definition (and understanding of the network of concepts) through which we pick out the phenomenon in the scientific domains *outside* of consciousness studies. As such, the phenomenon is a (theory-neutral) label we deploy for some state of affairs in the world (purportedly connected to a theory). On this view, the set of proposed empirical evidence of a theory (e_1_…e_n_), is a collection of phenomena purportedly connected to the theory. Next, how does this notion of e_1_…e_n_ impact the likelihood? Traditionally, the likelihood is the probability of the evidence *given* the hypothesis. But in our case, there is no straightforward entailment relation from the evidence to the theory (hypothesis). Currently every theory is (radically) underdetermined by the evidence. Consequently, another connection is needed between the hypothesis and the evidence. One possibility is to conceive of the connection along the lines of a probability that the evidence is *as* the hypothesis explains it. In other words, the likelihood is the extent to which the theory explains or predicts the phenomenon. The next section will unpack this to lay the foundation for the quantification of evidence (that will be discussed in section 4.4).

### 4.1 The likelihood: from arguments to ordinal rankings

In this section, the objective is to construct the Likelihood variable of Bayes’ theorem through the use of an intermediary ordinal categorization of a piece of evidence (each individual e in e_1_…e_n_). As a preliminary consideration, it is imperative to proceed from the assumption that the core principle (e.g., broadcasting in the workspace theories) and core concepts (e.g., overflow, and ‘rich’ phenomenality in recurrent processing theory) of a theory are valid when assessing evidence proposed of the theory. One reason for this is what is sometimes called *conceptual bleed*. Briefly, in order to make inferences for or against a theory from some empirical datum (the phenomenon) one needs, as a minimum, an interpretation that brings the concepts of the datum and the theory into a shared vocabulary; a kind of conceptual mapping. However, the conceptual mapping impacts (bleeds into) the possible inferences one can make from the observation(s) of the phenomenon. Furthermore, how one prefers to conceptualize and describe phenomena (the *intension* of the explanandum, i.e., consciousness) affects the mapping. This is a natural consequence of the conceptual and theoretical commitments of the researcher, who when interpreting relevant empirical data, will (reasonably) make use of the concepts she thinks best describe and categorize the phenomenon under investigation. Succinctly put, conceptual bleed means that commitments one has in the conceptual domain bleeds into, as it were, considerations and interpretations in the empirical domain. This makes it problematic to evaluate the proposed evidence for a theory “from the outside” (as it were), since the theory has bled into the evidence. One way to safeguard against this is to evaluate each theory on its own terms to avoid begging the question against its conceptual framework (as noted by Fazekas and me, see also above). Importantly, this does not mean that anything goes with respect to claims of empirical evidence. Previous work has shown errors that undermined proposed empirical support even assuming a theory’s conceptual framework. This is possible for instance by mischaracterizing the empirical data (e.g., D’Aloisio’s deployment of aphantasics’ performance on retro cue tasks, see Kirkeby-Hinrup and Fazekas, 2021 section 9) or in cases of unsound deductive arguments (Kirkeby-Hinrup, 2014).

Quantification then is the task of determining the value of a given set of evidence (e_1_…e_n_) which in turn requires determining the value of each piece of evidence (each individual “e” in the set of evidence proposed in favor of a theory),^19^ where “value” means “how much” a given *phenomenon* supports a theory (and “how much,” in turn, entails coming up with an actual number). Since there was no immediate way of coming to numbers directly from arguments based on observations, an intermediary element is needed to facilitate the translation. The rest of this section will develop a proposal for this intermediary element. The approach is to categorize arguments on an ordinal scale, which can then serve as an anchor for the actual quantification of evidence (discussed in section 4.4). Categorization of arguments essentially involves assessing them according to some criteria to determine their place on the ordinal scale. For ease of exposition, I will call the result of this assessment the “A-score” of the argument. In addition to facilitating the placement on the ordinal scale, such assessment serves to satisfy a prerequisite for any IBE process, namely determining whether the evidence does in fact support the theory. This is critical since, clearly, we should not count a piece of empirical evidence in favor of a theory unless it in fact supports the theory. So, initially, what is at stake here is whether the proposed connection between a piece of empirical data and a theory of consciousness is sound. Now, *if* the phenomenon can in fact support the theory (i.e., the argument is coherent), we want some gauge of the amount of support it can lend to the theory, i.e., to assess how *good* the argument is. But what exactly does “good” mean in this context?

Assuming that the argument is sound, and that other pitfalls are avoided (see Table 1) so we can say a phenomenon in fact supports a theory, there are two parameters we can deploy to assess how good a piece of support is. The first is theory-neutral vocabulary, and the second is testability. For instance, it is possible to mount a coherent argument that is nevertheless cached in the conceptual framework of a theory to an extent, where all the explanatory work is done entirely by the concepts of the theory, and there is no way to test the explanation without presuming the conceptual framework. In such a case, we would want to say that the phenomenon does in fact support the theory (because the argument is coherent), but it cannot lend very much support (because the argument is exclusively theory-dependent and untestable). Assuming that there is no smaller amount of support a theory can enjoy than a coherent yet untestable (and otherwise unworkable) argument, let us use this A-score category (“Coherent but untestable”) as the lower bound on our ordinal scale. From this, one can conceive of the next category as merely modifying the testability. The question here is whether the interpretation, or any part of it, is testable (in principle) without presuming the conceptual framework of the theory. Consequently, let us call the second ordinal score “Coherent and testable”. In the final category (A-score), let us collect the evidence that is not only testable in principle (without presuming the conceptual framework of the theory), but has *in fact* been tested. This leaves us with an ordinal ranking of claims of empirical support of four categories (A-score): Accepted, Coherent and testable, Coherent but untestable, and Rejected (Table 1).

**Table 1:** The A-score.

Rejected	The *phenomenon* is incorrectly represented and/or the interpretation of the *observation* is faulty and/or the *argument* based on the interpretation is not sound.
Coherent but untestable	The concepts deployed in the interpretation of the *observation* do all the explanatory work. There is no way to test the interpretation — using the exact same empirical *phenomenon —* that does not rely on presuming the theory and/or concept.
Coherent and testable	The interpretation of the *phenomenon* is testable in principle without presuming the entire theory and/or all concepts deployed in the interpretation.
Accepted	The phenomenon has been tested and the argument is sound, and both align with the central principle of the theory, or the defended concept.

### 4.2 The marginal: from phenomena to ordinal rankings

Traditionally, the marginal in Bayes’ theorem is cashed out as “the probability of the evidence,” but how should this be understood in the present context? Given that we do not have access to any/the objective probability of the evidence (the empirical phenomena claimed in support of a theory), we will again deploy ordinal scores as anchors for quantification. For the A-score we assessed arguments and how much these relied on the conceptual framework of a theory with respect to testability, but neither of these fit well as anchor for a (theory independent) *probability of the evidence* (the *phenomenon*). There are however good candidates for anchors for the marginal inherent in the observations of the phenomenon itself. Here, I will focus on one possible candidate, namely: replication. Initially, three things are worth mentioning with respect to the notion of replication as deployed here.

Firstly, we can note that for every phenomenon there will be a number of replications of a given finding about it. Sometimes, (in case it is a rare or very new study), the number of replications will be zero, and the original finding constitutes the only report of the phenomenon. Now, given that non-existing findings cannot form the bases for claims of *empirical* support, the lowest amount of credence we could have in a phenomenon would then be a single finding that has not been replicated. Secondly, it is worth noting that, plausibly, replication should co-vary with *credence* (discussed in Section 3.1 above), given that we agree that well replicated findings, and well understood phenomena are more credible as evidence. Thirdly, replication allows many values, which in turn allows for multiple ordinals. This makes replication suitable for grouping into different ordinals that can then be used as anchors for quantification.

With these three things in mind, the questions then are: how many ordinals should there be? What should they be? And how do we scale the number of replications of a phenomenon to a category on the ordinal ranking? In the examples below, I will operate with a three-step ordinal ranking categorizing phenomena into “high,” “medium,” and “low” replication (the R-Score). However, given *arbitrariness* discussed above, the number of ordinal rankings should not be up to me, therefore my use of three categories in the examples below is exclusively to keep the example data simple and easy to read, and should not be taken to signal that these are (arbitrarily) set in stone. While my inclination is to think that a relatively small number of ordinals such as this (or 4 in case one prefers a category for single cases with no replication) is most reasonable (and I suspect investigations will find convergence on this, similar to how the PAS scale was developed), nothing in the following hinges on this; there could be arbitrarily many categories (we could create a category for the exact number of replications of each phenomenon) since the methodology deployed in the quantification can accommodate this. For the present purposes, the working assumption merely is that we can meaningfully sort phenomena into three categories reflecting amounts of replication that we call “Low,” “Medium” and “High,” leaving the exact Low-Medium, and Medium-High thresholds unspecified. Nevertheless, because there may be disagreements between researchers (e.g., due to conceptual bleed) pertaining to selection, ordering, and assignment to the ordinal categories, the *arbitrariness* issue in this domain needs to be dealt with. In section 4.6 below, I consider ways to deal with this.

### 4.3 The prior and scaling

The last element of Bayes’ theorem we need to account for is the prior. Traditionally, the prior is the initial probability of the hypothesis (i.e., the theory). Given that the conceptual debates have come up inconclusive, it seems that assigning a higher initial probability (prior) to one or the other theory would be arbitrary. One way to avoid this is to assign the same initial probability (prior) to every theory. This also reflects the fact that we — as a field — really do not *know* which theory is right.^20^ But which value should it be set to? Normally, if we did not know either way, we would set the prior to 0.5 (50%). However, because there are multiple competing theories, the question is not exactly an either-or (fifty-fifty) proposition. An alternative would be to divide full confidence (100%) by the number of theories available. The number of contemporary theories varies between reviews (Northoff and Lamme, 2020; Sattin et al., 2021; Signorelli et al., 2021; Seth and Bayne, 2022). In the examples below, (as a conservative choice) the count is set at 25, and consequently 0.04 priors are used in the example data. Importantly, with respect to the comparison, as long as we stay impartial by assigning the same prior to each theory, the exact number of the prior is inconsequential. However, from a Bayesian perspective the lower and upper bounds are 0 and 1, respectively. So — if one desires to stay within a Bayesian framework — this constrains the scaling, given that no posterior of any theory should end up outside these bounds (<0 or > 1) at any point in the quantification process. Similarly, for comparison purposes — as long as we stay neutral and deploy the same values in the quantification of support for each theory — the exact scaling we deploy in the updating function is inconsequential. However, to stay within a Bayesian framework it is desirable that the amount of credence a phenomenon can maximally lend to a theory (the Likelihood) is not such that any individual quantified phenomenon, or the total set of phenomena takes the posterior above one or below zero.^21^

### 4.4 From ordinals to numbers

**Figure 3:**
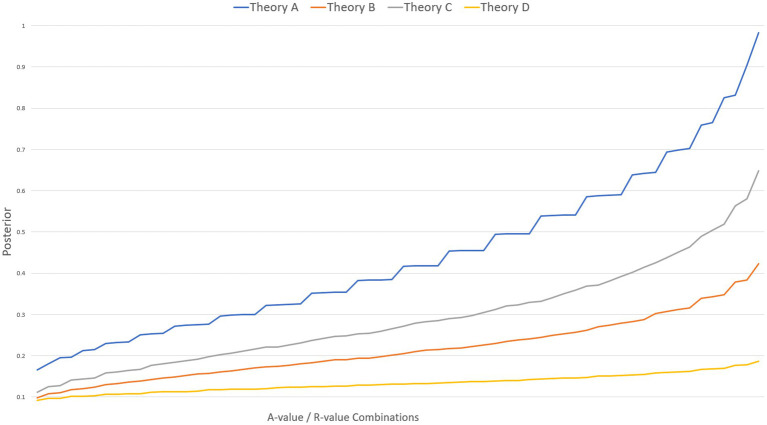
Impact of middle ordinal combinations (x) on posterior (y).

In this section, the topic will be how to get numbers from the ordinals (A-scores and R-Scores). To avoid confusion, I will deploy the terms A-value and R-value to signify a given number derived from a specific ordinal score. The central idea in QBE is to deploy the ordinal scores as anchors to provide natural minimum and maximum values (with one or more values of the middle ordinal(s) between). To illustrate: The A-score deploys the categories “Accepted,” “Coherent and testable,” “Coherent but untestable,” and “Rejected.” Not counting rejected evidence, we end up with a three-step ordinal where “Accepted” is better than “Coherent and testable” and both are better than “Coherent but untestable.” The highest ordinal (“Accepted”) is deployed as the natural maximum A-value we would assign in the quantification. Similarly, “Coherent but untestable” is the natural minimum A-value, being the lowest ordinal. As mentioned in the previous section, the exact scaling of the A-value in the updating function is inconsequential, as long as we deploy the same scale for each theory being compared. In the examples here, I will deploy a scale of 1–10 % for the A-values, meaning the lowest increase in posterior a theory can gain from a piece of evidence is 1%, and the highest gain is a 10% increase. Given that the A-Score “Accepted” is the natural max, that means that a piece of evidence with the A-Score “Accepted” should increase the posterior by 10 % (the A-value is 10%). Similarly, a piece of evidence with the lowest ordinal A-Score (“Coherent but untestable”) should increase the posterior by 1 % (the A-value is 1%). In this way the highest and lowest ordinals anchor any scale we decide on. But how do we non-arbitrarily set the A-value of the middle ordinal(s)? Disagreement seems possible on this question. For instance, one might suggest that the A-value of the middle ordinal should be in the middle (or close to) between the min and max, say 5 % on the scale used here. Others might disagree and argue that the testability difference between “Coherent and testable” and “Coherent but untestable” is of such significance that the A-value of the middle ordinal should be closer to 8 or 9 percent, rather than in the middle. So, how do we determine what the A-value of the middle ordinal (e.g., evidence scored as “Coherent and testable”) should be? Critically, we need to deal with *arbitrariness* and not bias the comparison against any theory. This is especially important because the A-value of the middle ordinal influences the posteriors (because it determines the increase a theory gains from a piece of evidence with the A-Score “Coherent and testable.” see Figure 3). In QBE this issue is solved by refusing to fix the middle ordinal to one value. Instead the idea is to calculate the entire dataset for each possible value of the middle ordinal (e.g., using natural numbers 2 through 9 in our example data) and let the collective set of posteriors form the basis for our comparison of theories. The same solution is applied to the R-score. The example data here uses R-values of one to ten,^22^ and calculates the dataset with each possible R-value for the middle ordinal (“Medium Replication”). Consequently, in our example here, the output of the quantification for a given theory is a set 64 posteriors (8*8) reflecting each combination of the possible middle ordinals of the A-value (2–9%) and the R-value (2–9).

But what about a case where someone wants to deploy more than three ordinals? In these cases there will be two (or more) middle ordinals rather than one. While this increases the combinations in terms of the number of posteriors that need to be calculated, there is nothing inherently problematic with this. Naturally, because two or more middle ordinals are ranked *qua* ordinals, they constrain each other in terms of the values each can have. To illustrate, using a 1–10 scale, if the lower of two middle ordinals has a value of 4, this constrains the possible values of the higher middle ordinal to the numbers [5,6,7,8,9]. In sum, the methodology can easily accommodate cases where more than one middle ordinal is deployed. While the number of posteriors that will be calculated for a theory will increase with the number of middle ordinals, this increase is trivial, and not such that it poses a problem for the methodology.

### 4.5 Quantification and comparison

The objective in this section is to demonstrate the proposed methodology. Initially, as just discussed the exact scaling of the parameters is inconsequential for the comparison as long as it is applied to every theory being compared.^23^ Unfortunately, there is an immediate obstacle to the demonstration in that there are no datasets on which to demonstrate the methodology. We simply do not have a full view of all — and which — phenomena are claimed in favor of any theory (let alone all theories). While some work has been done on this (Kirkeby-Hinrup and Fazekas, 2021), there is considerable way to go before we have complete compilations for every theory, and have separately assessed each proposed piece of empirical support to derive an A-score and R-score (and any other score we may think of, see below). To address this issue, I wrote a small piece of software^24^ that generates random datasets for hypothetical theories. The datasets were set to contain between 20 and 40 phenomena, that each had an A-score and an R-score. Generation of A-and R-scores was weighted to output comparatively fewer of the highest ordinal (see footnote 20) to account for the fact that there currently is not much knock down evidence. I generated four hypothetical theories [A,B,C,D], whose resulting datasets sorted in 3 (A-score) by 3 (R-score) matrices are shown in Table 2. I then ran the updating mechanism (using a scaling that allowed the posteriors to stay within Bayesian 0 and 1 bounds) on the datasets of the four hypothetical theories.

**Table 2:** Hypothetical datasets of theories A, B, C, and D.

		Accepted			Coherent and testable	Coherent but untestable
	**Theory B**	A-score
R-score	High	1	0	4
	Medium	1	7	8
	Low	0	2	3
	**Theory B**	**A-score**		
R-score	High	0	2	1
	Medium	0	6	3
	Low	0	4	4
	**Theory C**	**A-score**		
R-score	High	0	2	0
	Medium	0	5	8
	Low	1	5	4
	**Theory D**	**A-score**		
R-score	High	1	1	1
	Medium	1	1	3
	Low	0	3	3

**Figure 4:**
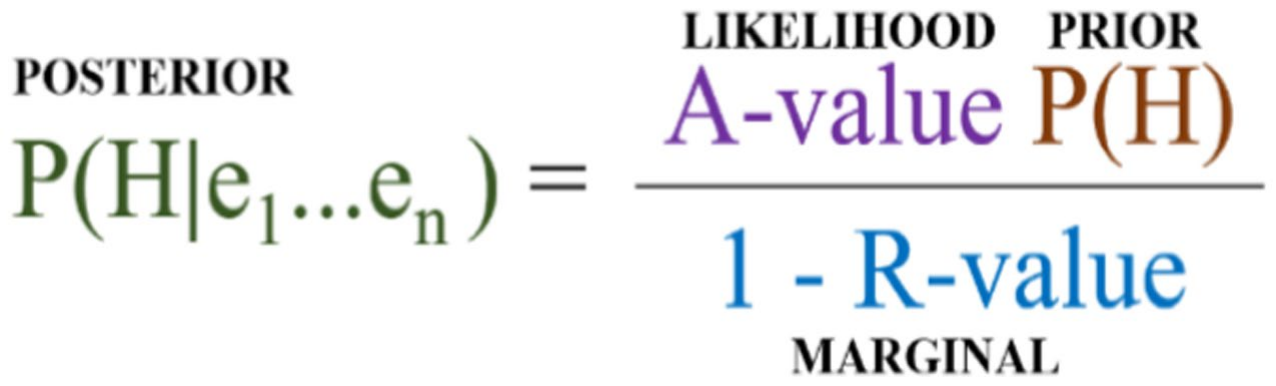
Example updating mechanism.

For determining the likelihood, a scale for the A-value of one to 10 % was used, meaning the highest A-score ordinal (“Accepted”) implied a 10 % increase, and the lowest ordinal (“Coherent but untestable”) a 1 % increase, with the middle ordinal (“Coherent and testable”) occupying each of the intermediary steps (2–9 percent). The marginal also used a one to ten scale for the R-value. However, the one to ten scale of the R-value was not percent, but rather hundreds. Again, the highest R-score ordinal (“High”) being 10/100, the lowest (“Low”) being 1/100, and the middle ordinal (“Medium”) occupying the intermediate steps (2–9/100). To avoid the counterintuitive result that well replicated studies might decrease a posterior, the marginal was calculated as one minus the R-value (Figure 4). The initial prior was set at 0.04 (based on the assumption that there are at least 25 viable competing theories). The updating itself consisted in iterating through the list of proposed evidence (phenomena) deriving a new posterior after the inclusion of each phenomenon, and that posterior becoming the new prior when updating with the next phenomenon. The end result, with the entire set of proposed evidence processed, is a posterior *given all the evidence*.

**Figure 5:**
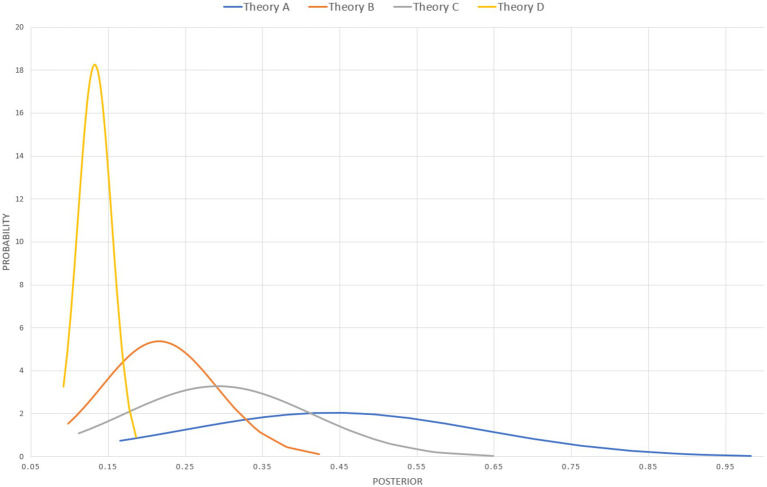
Normal distribution of probability (y-axis) that a theory has a given posterior (x-axis).

Now, because of the way R-values and A-values of the middle ordinals are modeled in QBE the updating has to be carried out with each of possible combination of R-value and A-value. In this example, the result after updating is a dataset for each theory consisting in sixty-four posteriors; *viz* one for each tested combination of values of the middle ordinal of the A-score (2–9%) and R-score (2-9/100). When ordering the datasets according to size of the posterior and plotting them on a graph, (Figure 3) the impact of the value of the middle ordinals of the A-score and R-score is evident. Similarly, by calculating the mean and standard deviation of all the posteriors (Table 3) we can represent the probability that a theory has a given posterior (Figure 5). While helpful — at a glance — to get an impression of where each theory stands, these are merely ways of depicting the data, and do not suffice as an actual *comparison.*

**Table 3:** Means and standard deviation.

	A	B	C	D
Mean posterior^*^	0.4409	0.2157	0.292	0.1329
Standard deviation^*^	0.1940	0.0744	0.1218	0.0219

^*^Rounded to 4 decimals.

There are several different ways of going about comparing the numbers. I will next consider a few options. The most straightforward approach would be to compare directly the mean posteriors of the theories, i.e., collapse the set of posteriors from each theory into a mean posterior for each theory and compare them. The mean posteriors — in themselves — allow for straightforward comparison of the theories on the bases of their respective posteriors (Table 3).

**Figure 6:**
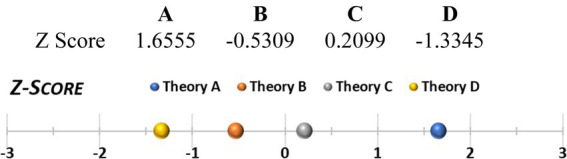
Z-score comparison.

A similar second option could be to take the graphs in Figure 3 and compare the areas under the curve (this could be refined using smaller increments for the calculated A-and R-values and by deploying integrals). However, a more interesting third option may be Z-score comparison (Figure 6). The idea behind Z-scores is to use the mean and SD of posteriors of all theories to create an anchor for how much support a given theory has as compared to the average support theories have. The Z-score shows how many standard deviations a theory’s support is from the average. Ranging from-3 to +3, positive Z-scores indicate good support relative to the norm. For comparisons of two concrete theories, a fourth option could be deploying t-tests, either one-tailed (pairwise comparison), or two-tailed, to assess whether the empirical support for theories across the field is truly different. Finally, one could compare theories directly against each other using pairwise ratios of the mean posteriors (Table 4).

**Table 4:** Pairwise ratio comparison.

	A	B	C	D
A (mean 0.4409)	X	2.0442	1.5099	3.3175
B (mean 0.2157)	0.4892	X	0.7387	1.6229
C (mean 0.2919)	0.6623	1.3538	X	2.1971
D (mean 0.1290)	0.3014	0.6162	0.4552	X

One important feature of each of these comparison options is that they are all independent of our scaling choices in the sense that — while it is nice that our data fits within the Bayesian bounds — the comparisons themselves do not depend on this (i.e., we could still do mean posteriors, Z-scores, ratio comparisons etc. if the posteriors were higher than 1). This gives significant flexibility to our choices with respect to scaling, and counters potential issues with arbitrariness in this regard.

### 4.6 Arbitrariness in scoring, ordinals, and the updating mechanism

In the above, *arbitrariness* has been prevented at every turn, yet three issues remain in this regard that need to be addressed. The first (and most critical) of these is *who* gets to determine the A-score of a piece of proposed empirical support. Given that the A-score directly impacts the amount of support gained from a piece of evidence, if this is left at the whim of the comparer, the whole process is undermined. The solution is straightforward: in the scoring it is necessary to engage with the original authors of a given piece of proposed empirical support.^25^ Such engagement serves to make certain that the A-score assigned to each piece of empirical evidence is corroborated by the views of the original authors.^26^ Furthermore, the engagement with the proponents of a given piece of empirical support affords them opportunity to clear up misunderstandings, make corrections, or further specify their argument in light of problems exposed (that result in an A-score they disagree with), or questions that arose in the case-by-case analysis. It is also important to recognize that novel experimental paradigms may impact A-scores. One recent example of this pertains to the pneumatic drill example given in favor of the distinction between access consciousness and phenomenal consciousness (Block, 1998). The gist of the ‘pneumatic drill effect’ is that upon the disappearance of an (previously un-accessed) auditory source, subjects have a strong intuition that they had been experiencing it all along. By virtue of being a ‘dishwasher example’ (see Kirkeby-Hinrup and Fazekas, 2021, p. 6 for details), previously this would have been classified as ‘Coherent but Untestable’. However, a novel study (Amir et al., 2023) operationalized the same effect with results seeming to corroborate the intuition. Consequently, the pneumatic drill effect more properly belongs in the ‘Coherent and Testable’ category.

Being sensitive to novel findings and engaging with the proponents of theories in this way means that the conclusions reached will properly reflect the views and data in the field, and the datasets deployed in the comparisons are accurately reflect the evidence out there and are broadly endorsed. Accusations of arbitrariness in the scoring of evidence are catered to by allowing proponents of theories to spell out the reasoning behind a given piece of proposed evidence, spell out potential testability, or provide updates to arguments. Following any changes to A-scores as a result of such interaction, re-scoring the piece of evidence and re-calculating posteriors is trivial.

The second issue pertains to *arbitrariness* in deciding the ordinal categories and the criteria for each category. To illustrate, whether one deploys a three-step ordinal or a five-step ordinal for the R-score impacts the posteriors of theories because if there is a larger number of ordinal steps this means that two pieces of evidence that are scored as equal on a three-step ordinal (e.g., both in the “Medium” R-score), may not end up in the same ordinal category on a scale with more ordinals (e.g., one may end up in “Upper Medium” and the other in “Lower Medium”). Consequently, since the R-score is the foundation of the R-value, which in turn impacts the posterior, the number of ordinals and their criteria impact the comparison and may bias the comparison against theories whose evidence ends up being ‘worth’ comparatively less if a higher number of ordinals is deployed. A similar problem pertains to the criteria for being scored in a given ordinal. To illustrate, whether 50 or 55 replications is the criterion for “High” replication (the highest ordinal) matters for phenomena with a number of replications between 50 and 54. So, how do we best settle on the ordinal categories, the number of ordinals, and their respective criteria? Again — for by now familiar reasons — no single individual should get to decide these questions. Therefore, it is useful to consider some possible ways of solving this issue.

The first way consists in letting the scientific community decide the categories and criteria. This could either be done as a straight-up crowdsourcing endeavor with questionnaires disseminated through appropriate channels (specialist mailing lists, conferences, websites, or journals), or in a more structured way. One example of such a process is the development of the Perceptual Awareness Scale (“PAS,” see Ramsøy and Overgaard, 2004; Overgaard et al., 2006) which found that when asked to compose their own scale for visual awareness, subjects’ responses converged on a four step ordinal. PAS is widely recognized as useful (it is probably by far the most deployed scale to assess perceptual awareness in contemporary consciousness science) and has (to my knowledge) never faced significant accusations of arbitrariness. Now, there is of course a sense in which a crowdsourced scale would be arbitrary to the sampled population (i.e., the ‘crowd’). However, this is not the arbitrariness of relevance here given that the sampled crowd is the scientific community, and these are exactly the people whose views we would want the scale to reflect.

The other possible way one could attempt to settle this is by deriving it though data mining the relevant academic body of work. This would consist in surveying the replication numbers of the phenomena to identify the ranges where clustering occurs, and then using the number of clusters to determine the number of ordinals, and the ranges of the clusters to determine the thresholds for a given ordinal. With respect to this kind of data mining, there are a wide range of established algorithms to determine not only the number of clusters in a dataset, but also the values of those clusters (e.g., k-means clustering and x-means clustering to give just two examples). So if we have a dataset containing the number of replications for all the proposed phenomena, we could derive the number of categories (ordinals) and the cut-offs between them.

The third issue concerns decisions about the updating mechanism. In the example above, I used a scale of 1 through 10 percent for the A-score, meaning the prior got multiplied by a number (the A-value) in the range between 1.01 and 1.1. However, there are obviously other ways one could structure such an updating mechanism. To give just one simple alternative: instead of using a multiplication function, one might simply use addition (i.e., by just adding the A-value to the prior). It is trivial that the choice between multiplication and addition matters,^27^ given that the cumulative effect of several multiplications favors theories with a higher number of proposed pieces of empirical support. This means an updating mechanism deploying multiplication is biased against theories with a low number of proposed empirical support.^28^ Similarly, the R-value in the example above was modelled as a number between 0.9 and 0.99 (with the highest ordinal being 0.9) to achieve the effect that higher replication scores increased the amount of support a theory received from a phenomenon (because dividing by 0.9 yields a higher posterior than dividing by 0.99). However, there are a multitude of alternative ways in which one could model the R-score in the updating mechanism. One possibility is to use percentages like in the A-value and simply factor the R-score in with the likelihood along with the A-value (this matters because dividing by 0.9 is not equal to multiplying by 1.1). Furthermore, as I will touch on below, one might want to include more elements in the updating mechanism than arguments and replication. In sum, there are many ways to structure the updating mechanism, it is unclear which is preferable, and choosing between them runs the risk of arbitrariness. Certainly, involving mathematicians (especially statisticians) and philosophers of science would be beneficial to map out different possible updating mechanisms and clarifying their respective implications. In any case, I do not purport that the version presented above is anything more than an early sketch. In fact, I think it is incomplete in the sense that my (arbitrary) preference would be to expand it to account for more features of the evidence in the marginal (I will briefly return to this in the concluding remarks below). Now, given the centrality of the updating mechanism to QBE, it may seem as if *arbitrariness* in this place effectively subverts the whole idea. For instance, it is not an implausible scenario that disagreements about how to structure the updating mechanism may result in multiple competing versions, each with its own group of proponents and no non-arbitrary way to decide which version is preferable. In this case, it may appear that we are back where we started, and QBE has not managed to move forward the debate in any meaningful way. Fortunately, this appearance is misleading. The important progress to notice in this respect is that disagreeing about the updating mechanism is significantly different from disagreeing about the nature of consciousness. One way in which it is different is that discussions on the updating mechanism can be done objectively, in the sense that the subject matter is mathematics (statistics). This means QBE manages to cauterize the conceptual bleed from the theoretical predilections of researchers with respect to consciousness. Put differently, disagreement about the updating mechanism is an *entirely different debate* that can be had without utilizing any of the concepts the disagreements on which were at the root of our problems in ICS.

## 5 Concluding remarks

Proponents of competing theories of consciousness have spent the better part of almost three decades amassing empirical support for their preferred theory on the assumption that this would somehow resolve the debates. In recent years proposals specifically on *exactly how* empirical support can achieve this have garnered attention.

The approach I have advanced here offers a novel methodological approach to this issue. Throughout I have endeavored to be transparent about the fact that this is not a finished or unproblematic methodology, and that there are several avenues open for future development and refinement. QBE is merely a first approximation of the methodology. Its purpose here is more of a proof of concept that it is possible to quantify empirical support for theories of consciousness in a way that avoids *arbitrariness*, than a fully baked cake. In other words, at this stage QBE is not purported to be either perfect, or entirely noncontentious. Firstly, there may be additional ways of scoring evidence that could either complement or supersede the way proposed here. Secondly, there likely are unexplored ways to quantify the scores. Thirdly, there are many possible ways to construct the updating function in Bayes’ theorem. Each of these three avenues of development comes with separate requirements for justification of why it is preferable to other ways of doing the same thing. Or, if full justification is not possible, then the requirements can be for motivation, argument, or rationale, depending on one’s position on a range of philosophy of science issues, and one’s epistemological commitments. The proposal offered here is open to exactly that; i.e., that there may be better^29^ ways to model scoring, conversion, or updating, and the future development of the methodology should be open to change. The modest aim here has been to show that there *is* a model we can develop.

One way to develop the model could be to construct additional ordinals. For instance, with respect to the Marginal in Bayes’ theorem, the three remaining aspects^30^ of the two drivers of the intuition discussed in section 3.1 provide avenues of development.^31^ For instance, it might be relevant to introduce ordinals to score phenomena in accordance with the two aspects of the *closeness* driver. This would mean phenomena would also be scored according to physical/functional closeness (e.g., with categories such as computer models, animal studies, human studies) or Distribution (e.g., with categories such as: Single case, Rare, Common, Prevalent). Similarly, one may want to introduce an ordinal to reflect the other aspect of the *credence* driver (scope). Naturally, each new ordinal one introduces brings a demand for considerations about how this ordinal is then best implemented in the updating mechanism. One strength of QBE is that revisions of both the datasets, scoring, quantification, and updating mechanism are easily handled, which serves to underscore the objectivity, and flexibility of the methodology.

Finally, more should be said on how my QBE avoids the shortcomings of the two current approaches for comparing theories of consciousness, namely the adversarial collaboration (ARC) and criterion-based (CRIT) approaches (As discussed in Section 2). For each of ARC and CRIT, I identified four issues and in Section 3.1, I argued that it was desirable if QBE could avoid these issues. Therefore, a brief summary of how QBE manages to do this is warranted. Firstly, there is no upper limit for the number of theories to which QBE can be simultaneously applied. This means that the issue of *targeted theories* does not pertain to QBE. By considering all available evidence QBE has the broadest possible scope, thereby avoiding the *narrow scope* issue. Similarly, in QBE, the same methodology is applied to all theories thereby avoiding the issue of *generalizability*. The methodology in QBE allows for easy addition, removal, or updating of theories or evidence. This means that the *robustness* issue is also avoided. While the process of collecting and scoring all empirical evidence proposed in favor of every theory constitutes a significant amount of work, it is a one-time effort, in the sense that once the datasets are collected, updating them with further proposed evidence is trivial. This means that while the initial *cost* of QBE is somewhat high, it is nevertheless significantly less than that of ARC (both in the short and long term). Because of the Bayesian updating process, QBE is sensitive to every piece of evidence proposed in favor of a theory. Consequently, by accounting for the total amount of evidence, QBE avoids the *sensitivity* issue. With respect to the *arbitration* issue, the scoring and updating process in QBE make ties highly unlikely. Furthermore, because QBE allows for easy updating, ties will be broken upon the addition — or revision — of a single piece of evidence. QBE is maximally flexible by allowing for any piece of proposed evidence to be scored and added. Thus the *flexibility* issue is also catered to. Finally, at every point it has occurred, I have addressed the *arbitrariness* issue with respect to the topic at hand. Most importantly, by generating sets of posteriors for each theory based on every possible scoring of the evidence, no decisions about evidential weight depend on the judgment of any individual.

Having argued that QBE avoids the issues identified with ARC and CRIT, it is necessary to reiterate that the objective of QBE is not to supplant these two approaches, but to offer a third approach, to be deployed either independently of — or jointly with — ARC and CRIT. In other words, the different approaches need not be mutually exclusive, but rather may in fact positively interact. For instance, prognosis output from QBE may inform ARC work by indicating relevant theories to test against each other. Reciprocally, findings from ARC projects may be scored and added as evidence in QBE. In a similar vein, CRIT contains meta-theoretic considerations (e.g., regarding what we want theories to explain) that have merit on a general level. It seems there is not only room for co-existence, but also for synergy between the different approaches to assessing and comparing theories of consciousness.

## Data availability statement

The raw data supporting the conclusions of this article will be made available by the authors, without undue reservation.

## Author contributions

AK-H: Conceptualization, Formal analysis, Methodology, Software, Visualization, Writing – original draft, Writing – review & editing.

## Footnotes

^1^Accelerating research on consciousness: an adversarial collaboration to test contradictory predictions of Global Neuronal Workspace and Integrated Information Theory; https://www.templetonworldcharity.org/projects-database/accelerating-research-consciousness-adversarial-collaboration-test-contradictory. The sizable grant this project received from the Templeton Foundation speaks to the growing recognition that there is a need for new and ambitious approaches to assessing and comparing theories if we are to make progress in interdisciplinary consciousness studies.

^2^Where “kind” can be understood either as type of evidence or as strength of evidence.

^3^https://www.templetonworldcharity.org/accelerating-research-consciousness-our-structured-adversarial-collaboration-projects for further info.

^4^Three in the first project (Ferrante et al., 2023).

^5^Observe that in a situation where no one ever changes their mind, it is useful to be able to assess overall plausibility because this allows us to still say something about which theories are preferable.

^6^This methodological issue is — of course — not unique to ARC.

^7^There are already candidates for further criteria (Overgaard and myself have proposed one Kirkeby-Hinrup and Overgaard, 2023).

^8^This is tenuous of course, given that there may be a range of other reasons elements were not included in a paper.

^9^Assuming it would not make sense to add a criterion that every theory satisfies.

^10^Another plausible reason is the lack of datasets necessary to carry out IBE, see Section 4.

^11^We suggested the first stage — in which proponents of theories collected empirical support — had already been ongoing for a couple of decades, and therefore there was no need to demonstrate its feasibility.

^12^Given the complexity of debates between competing theories of consciousness, it is not sufficient to select reports in which empirical evidence is proposed explicitly in support of a theory. In fact, significant (and increasing) parts of the academic exchange between proponents of competing theories take place in so-called proxy debates. Proxy debates, as the name indicates, are not directly about the theories. Instead, they are about specific empirical phenomena or aspects of consciousness, on which the positions taken in the debate are (sometimes tacit) extensions of central views of particular theories. Therefore, when proxy debates deploy empirical evidence, this should be seen as part of the empirical support for a theory. Over the last couple of decades the occurrence of such proxy debates has been relatively steady. Examples include the debates about unconscious perception (Brogaard, 2011; Block, 2016; Peters et al., 2017), non-conceptual content (Brinck, 1999; Jacobson and Putnam, 2016), whether perception is rich or sparse (Kouider et al., 2010; Block, 2011b, 2014b; Knotts et al., 2019), and perceptual precision (Block, 2014a; Prettyman, 2019). A prominent ongoing proxy debate concerns the localization of the neural correlates of consciousness (usually called either the “front vs. back” debate or the “early vs. late” debate). For a small sample of this debate see, e.g., (Lamme, 2003, 2004; Bor and Seth, 2012; Meuwese et al., 2013; Frässle et al., 2014; Kozuch, 2014; Boly et al., 2017; Odegaard et al., 2017; Michel and Morales, 2020).

^13^While the methodology proposed here caters to the fourth step and thereby complete the account offered by Fazekas and me, nothing in the below hinges on acceptance of our claims in that paper. Readers uninclined to this view of an IBE process may nevertheless find use for a methodology for quantifying empirical evidence and comparing sets of evidence.

^14^The rest will be addressed in the concluding remarks.

^15^Observe, I do not claim that *closeness* and *credence* are the only possible sources for an intuition that some instances of empirical support should be given more weight than others when comparing theories of consciousness. It is entirely possible that there are other sources for this intuition. Nevertheless, since this intuition appears to be rooted at least in a combination of our areas of interest (*closeness*) and scientific principles (*credence*), I will assume we can agree on one or more of the drivers *physical/functional closeness, distribution, replication, and scope*. Importantly, my proposal does not depend on whether the reader subscribes to this intuition (or all of its possible drivers). QBE can be deployed independently of this intuition (for instance as a feasible theory neutral way to assess and compare theories of consciousness, that does not have the shortcomings of CRIT and ARC).

^16^Relevant questions in this regard include whether the same concepts are applied to — or operationalized in (Fink, 2016) — the interpretation in a uniform way. Whether there are equivocations or vagueness in the application of terms from the conceptual framework. It is crucial correctly to identify the theoretical claim defended, since this has implications for the way the argument is evaluated. Plainly, to assess an argument it is imperative to identify what it is an argument *for*. It matters for the assessment whether an argument is about overflow or recurrent processing (even if both pertain to RPT). To understand an argument in this context is to investigate how the proposed interpretations of the empirical evidence map onto the theoretical principle or concept, and illuminating (by extrapolation, if necessary) the argument connecting the interpretation of the empirical evidence to a theoretical claim. Depending on the principle/concept defended and the empirical evidence, the argument may take various forms. In some cases, a claim of empirical support deploys two or more empirical phenomena interwoven into a complex argument that requires careful analysis before it can be assessed (see, e.g., Brinck and Kirkeby-Hinrup, 2017). In other cases a concept is straightforwardly deployed to explain an empirical phenomenon, but even then, it is important to clarify all the details of the argument. What part of the argument is doing the explaining? Is the explanation reasonable? Are there any errors in the premises or the conclusion? Is there any vagueness or equivocation in the premises or conclusion? Is the interpretation of the empirical phenomenon true to the original empirical reports of the phenomenon? Is there any way we can test empirically whether the explanation offered by the theory is correct? Is the original empirical report framed directly in relation to the theory, or is it given an explanation post-hoc using the theory’s framework?

^17^A strength of QBE is that it concretizes the support of theories and simplifies comparison (numbers are easily graspable and easy to compare).

^18^Part of which involves concepts proprietary to the framework of a theory.

^19^Thus, the process here is to consider the arguments connecting each proposed piece of evidence (each phenomenon) to the theory of consciousness whose empirical support we are evaluating. This entails assessing the proposed evidence for each theory on a case-by-case basis (explication and examples of this process can be found in Kirkeby-Hinrup and Fazekas, 2021).

^20^Perhaps priors could be modified to reflect the simplicity of a given theory, appealing to Occam’s Razor, with simpler theories being assigned a higher prior. I am somewhat skeptical of this implementation. On the one hand, I think simplicity is secondary to aspects such as explanatory power, coherence, and empirical support. On the other hand, there are several ways in which theories can be “simple” so this comes with an additional need to eliminate arbitrariness with respect to which aspect(s) of a theory the simplicity should apply to. Nevertheless, at this stage of the development in the model, it is certainly premature to rule anything out, perhaps there is both room and warrant for simplicity considerations somewhere in it.

^21^A further benefit of this Bayesian constraint on the bounds of posteriors is that it allows us to consider sets of posteriors as probability distributions, which will be relevant for the comparison step discussed in the next section.

^22^Again: arbitrarily chosen since the exact scaling is inconsequential for the comparison. The important thing is to keep it the same for all theories.

^23^In the previous sections, I have mentioned at the parameters that will be deployed here, but I will reiterate them the first time they appear.

^24^Not on github, but open source in the sense that I will give you the code if you send me an email.

^25^As well as other relevant proponents of the theory.

^26^Remember, their conceptual framework has to be taken for granted, which makes them the authorities on interpretation of their proposed evidence, as noted in section three.

^27^Or other possible ways of conceiving of the updating mechanism.

^28^One might argue that intuitively this makes sense given that large amounts of empirical support *should* result in higher credence in a theory, when compared to theories with very few pieces of empirical support. This, however, is a separate discussion, and I will leave it to the side for now.

^29^Where “better” can be understood in various ways. To give just a few examples, better could be understood in terms of justification, motivation, simplicity, appeal, alignment with common intuitions, more fine-grained, or more refined mathematically.

^30^The R-score already reflects the “Replication” aspect of the *credence* driver.

^31^Specifically, to my mind, these three remaining aspects each seem relevant to the Marginal in the updating mechanism.
